# Implementation of offset corrected AGAD algorithm on 130-nm CMOS technology–based RRAM array for analog neural network training

**DOI:** 10.1126/sciadv.aee7134

**Published:** 2026-09-25

**Authors:** Jimin Lee, Paul Solomon, Nanbo Gong, Omobayode Fagbohungbe, Russel Wilson, Takashi Ando, Seyoung Kim

**Affiliations:** ^1^ Department of Electrical Engineering, Pohang University of Science and Technology, Pohang 37673, Republic of Korea.; ^2^ IBM Thomas J. Watson Research Center, Yorktown Heights, NY 10598, USA.

## Abstract

We present the first implementation of the analog gradient accumulation with dynamic reference (AGAD), reported as the most advanced and highest-performing version of the TT (Tiki-Taka) algorithm, on an HfO_2_-based resistive random-access memory (RRAM) array for analog neural network training. Through comparative simulations with recent versions of the TT algorithm, we verify that only AGAD enables rapid and accurate gradient accumulation, even in the presence of reference errors. This robustness is enabled by the chopper technique and moving-average–based dynamic digital reference. For demonstration, we propose and fabricate a novel 4T1R unit-cell crossbar array that offers excellent retention while eliminating half-select disturbances. Notably, we experimentally implement the bidirectional weight update behavior of AGAD on hardware and extend it to an array-level weight transfer task, achieving successful weight convergence with a low loss of 0.005, essential for neural network training. This study emphasizes that AGAD can provide a viable pathway toward practical in-memory computing under realistic device limitations.

## INTRODUCTION

Artificial intelligence (AI) technologies have advanced rapidly, driven by substantial progress in deep neural networks (DNNs), which now serve as the backbone of applications such as autonomous driving, speech recognition, and large language models (LLMs) (*1*–*6*). To handle these AI workloads—which require both substantial computational power and frequent memory access—parallel architecture, most notably graphics processing units (GPUs), have emerged as the dominant solution to overcome the memory bottleneck inherent in the traditional von-Neumann architecture. While GPUs offer outstanding computing performance at processing large volumes of data, they suffer from high power consumption, prompting increasing interest in more energy-efficient digital accelerators such as field-programmable gate arrays (FPGAs) and application-specific integrated circuits (ASICs) (*7*–*11*).

**Fig. 1.**
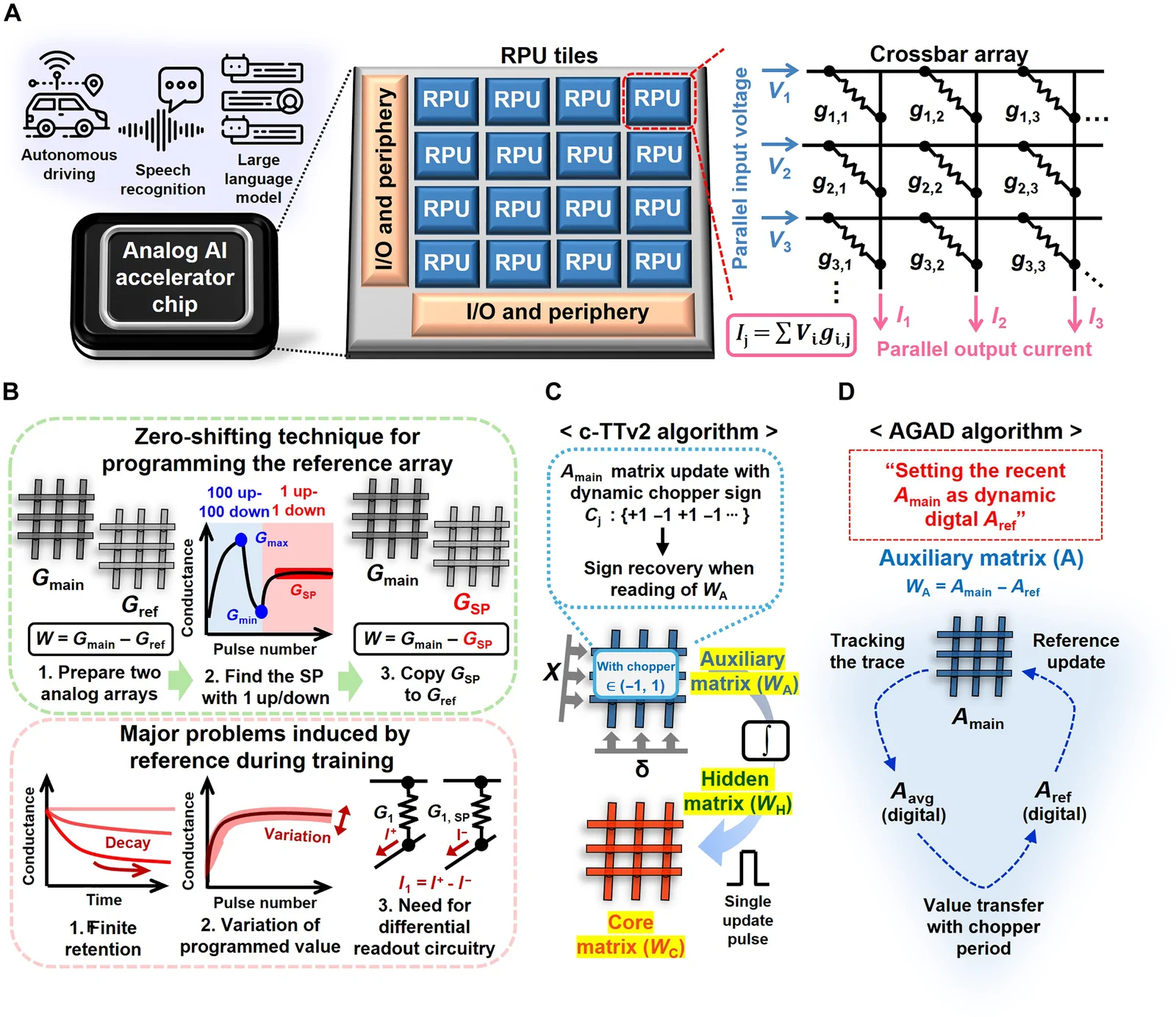
Overview of an AIMC system and algorithmic framework for training in nonideal crossbar arrays. (**A**) Emerging neuromorphic system architecture of an analog AI accelerator chip with RPU based crossbar array and periphery circuit. (**B**) Procedure of zero-shifting technique and major challenges of reference programming. Schematic of (**C**) c-TTv2 and (**D**) AGAD algorithm. Icons in (A) were designed by kmg design (https://www.flaticon.com/free-icon/smart-car_6810569?term=autonomous&page=2&position=29&origin=search&related_id=6810569), Smashicons (https://www.flaticon.com/free-icon/voice-message_1599234?term=voice&page=1&position=14&origin=search&related_id=1599234), and Magnific (https://www.flaticon.com/free-icon/chat_12574772?term=chat&page=3&position=5&origin=search&related_id=12574772) from Flaticon.

In parallel, analog AI accelerators have garnered increasing attention due to their ability to compute directly within memory devices (*7*, *12*–*15*). Resistive processing units (RPUs) based on emerging nonvolatile memory (NVM) devices such as resistive random-access memory (RRAM), phase change memory (PCM), and electrochemical random-access memory (ECRAM) are being actively explored as promising candidates and can be expanded into crossbar array to support analog in-memory computing (AIMC) (*16*–*22*). This architecture leverages Ohm’s law and Kirchoff’s current law (KCL) to conduct matrix-vector multiplication (MVM) in a highly parallel and localized manner (Fig. 1A). Thus, it enables faster multiply-and-accumulate (MAC) operation, and superior energy efficiency compared to normal digital approaches. These advantages have been demonstrated in several recent works focusing on inference tasks using RRAM-based analog accelerators (*23*–*26*).

Furthermore, since the training phase has significantly higher computational load than inference, the training implementation of AIMC provides even greater computational benefits. Despite this potential, the inherent nonidealities of NVM devices hinder the high neural network training accuracy using optimization algorithms such as stochastic gradient descent (SGD). One major issue is asymmetric switching caused by mismatch between potentiation and depression, where unequal conductance changes result in reduced weight update accuracy (*27*). In addition, because of intrinsic time constant of the device, conductance decay over time leads to short retention period and poor weight stability. The limited number of analog states within the switching window further reduces the weight precision. Last, cycle-to-cycle (c-to-c) and device-to-device (d-to-d) variations degrade overall training performance (*13*, *28*). To mitigate these issues, hardware-aware training algorithms have been developed to compensate for the nonideal characteristics of memory devices (*29*–*31*).

Among these algorithms, the Tiki-Taka (TT) algorithm has been shown to enable neural networks trained on nonideal devices to achieve performance comparable to those trained with ideal hardware (*29*, *32*, *33*). In particular, the Tiki-Taka algorithm version 2 (TTv2) demonstrated that training is feasible even with tens of states, showcasing the robustness under highly restricted device conditions (*33*–*38*). More recently, Chopped-TTv2 (c-TTv2) and analog gradient accumulation with dynamic reference (AGAD) algorithms have been proposed as state-of-the-art extensions of the TT algorithm (*39*). Both are designed to tolerate inaccuracies in reference conductance programming, and their converged training accuracy exceeds that of TTv2 using the chopper technique. Despite their promise, capabilities remain unverified due to the absence of both theoretical analysis and have not yet been implemented through experimental training. Therefore, evaluation with AIMC is crucial to validate the effectiveness of advanced TT algorithms.

In this paper, we demonstrate the neural network training of AGAD algorithm on an HfO_2_ based RRAM array. Our simulations and analysis with improved TT algorithms reveal that both the chopper technique and the digital reference derived from the recent state of the auxiliary matrix are necessary for the fast and accurate directional gradient computation, even in the presence of reference offset. To validate this in hardware, we design and fabricate a crossbar array architecture based on 4T1R unit-cell with 6-month superior retention characteristics, enabling local selection without half-select issue in adjacent cells. We further validate that the bidirectional weight update mechanism of AGAD on hardware operates robustly with asymmetric conductance modulation and under variations in the programmed reference. Building on these findings, we successfully implement array-level diagonal weight transfer with a low loss of 0.005, highlighting the applicability for high performance neural network training.

## RESULTS

### Offset correction of Tiki-Taka algorithm with chopper technique

A key objective of AIMC is to perform the learning directly within the array by accurately mapping synaptic weights onto analog devices and applying highly parallelized update schemes, supporting fast and efficient training. However, one of the fundamental challenges in analog weight representation arises because RPUs can only store positive conductance values. To encode both positive and negative weights, a differential readout is used where each weight is expressed as the difference between two conductance values, *G*_main_ and *G*_ref_. One method for setting the reference conductance values is the zero-shifting technique, which aims to align the reference conductance to a stable baseline (*40*). As illustrated in Fig. 1B, this technique involves three main steps: (i) preparing two analog arrays designated for main and reference conductance; (ii) locating the symmetry point (SP) *G*_SP_, starting with the identification of the switching window, as indicated by the blue-shaded region, followed by repeated 1 up/down pulses, as shown in the pink-shaded area; and (iii) copying the SP value to the reference array. In asymmetric devices, SP refers to a specific point where the conductance increment and decrement become balanced. This point must be initialized as the zero-value point to prevent weight distribution drift caused by inherent device asymmetry, which would otherwise degrade training performance. Despite its importance, the use of SP as a fixed reference during training introduces several practical challenges. One issue is finite retention, where the programmed reference gradually decays over time, leading to a mismatch between the intended and actual weight values. Another problem is the variation of programmed values across devices, which limits the precision of weight encoding and causes inconsistencies during gradient accumulation. In addition, it needs additional readout circuitry to compute the difference between the main and reference arrays, increasing hardware complexity and doubled chip area.

Meanwhile, one approach used for training is to carry out the outer product and weight update steps by leveraging coincidence of voltage pulse trains applied in parallel across rows and columns of the crossbar array—where the pulses along the row direction are proportional to the input activations, and those along the column direction are proportional to the error—thus achieving O (1) time complexity (*27*–*29*). When such a conventional stochastic gradient descent (SGD)–based stochastic weight update scheme is applied to devices with asymmetric weight update characteristics, the training process can fail. The device conductance tends to converge away from the target weight, reaching a balance point between the driving force toward the target and restoring force toward the SP (*27*). To address these hardware limitations, the TT algorithm proposes that replacing the weight array with two analog arrays—an auxiliary (A) matrix and core (C) matrix—which allows for separate handling of gradient and weight storage (*29*). Each of these weight matrices is paired with a reference array: $W_{A}=A_{\text{main}}-A_{\text{ref}}$, and $W_{C}=C_{\text{main}}-C_{\text{ref}}$. In the TT algorithm, the weight update is carried out on the A matrix, while the forward and backward pass are conducted using the C matrix. This separation effectively mitigates the impact of device asymmetry; it introduces noise during the transfer between two matrices and requires ∼1000 states. TTv2 resolves these challenges by incorporating a hidden (H) matrix that functions as a low-pass filter (*34*). It constantly accumulates gradient from the A matrix until a predefined threshold is reached. Once the threshold is exceeded, the H matrix resets, and a single update pulse is applied to the C matrix. This approach increases noise tolerance. However, the robustness of TTv2 is still sensitive to the accuracy of the programmed reference values (*39*).

Figure 1 (C and D) illustrates the c-TTv2 and AGAD algorithm using the chopper technique, and how it corrects the offset issue during gradient accumulation. A chopper technique, widely used to remove low frequency offset and 1/*f* noise in an analog circuit design, uses by modulating input signal to higher frequency, where the offset components are no longer dominant. After amplification, the signal is demodulated back to its original form, preserving the true signal while suppressing the unwanted offset (*41*). Inspired by this method, chopper technique is incorporated into the TTv2 during gradient accumulation in the *A*_main_ matrix. During the gradient update with the outer product of input X and backpropagation error δ, a chopper sign of either −1 or 1 is applied periodically according to the chopper probability ρ or randomly. Subsequently, when reading from the A matrix row-by-row, the original sign is recovered before the value in the *A* matrix is transferred to the *H* matrix. Then, the update is accumulated into the *H* matrix, thereby reducing the impact of an erroneous reference value. This mechanism can be further described mathematically by following equations

$$H_{t+2n}-H_{t}=\lambda k\left[o_{r}+g-\left(o_{r}-g\right)\right]=2\lambda \mathrm{kg}$$ (1)

where λ represents the learning rate and t denotes a specific time step. During the two chopper phases 2*n* with *k* reads, a constant gradient *g* is applied with opposite sign due to chopper modulation. The offset term $o_{r}$ remains unchanged across both phases. Therefore, the reference offset is canceled out, and only the gradient-dependent term contributes to the effective accumulated update. Nonetheless, large offsets require lowering the learning rate to suppress undesired weight changes, which in turn slows down the overall learning process. In response, the AGAD algorithm, which includes additional digital computation has been proposed. The AGAD is the most advanced TT algorithm, outperforming the c-TTv2 by introducing a digital reference to express the differential weight of the A matrix, which serves as a key enhancement. Unlike previous versions that rely on fixed reference value, AGAD dynamically adopts the most recent state of *A*_main_ matrix as a reference. This dynamic reference is updated through a leaky integration process, capturing the temporal average of weight update behavior rather than relying on instantaneous device responses. As a result, AGAD remains robust against d-to-d variations and nonidealities, such as asymmetric update characteristics or reference offsets, even in array-level implementations (*39*). By continuously adapting the reference to each device’s accumulated behavior, AGAD preserves the correct update direction regardless of variations in update magnitude across devices.

**Fig. 2.**
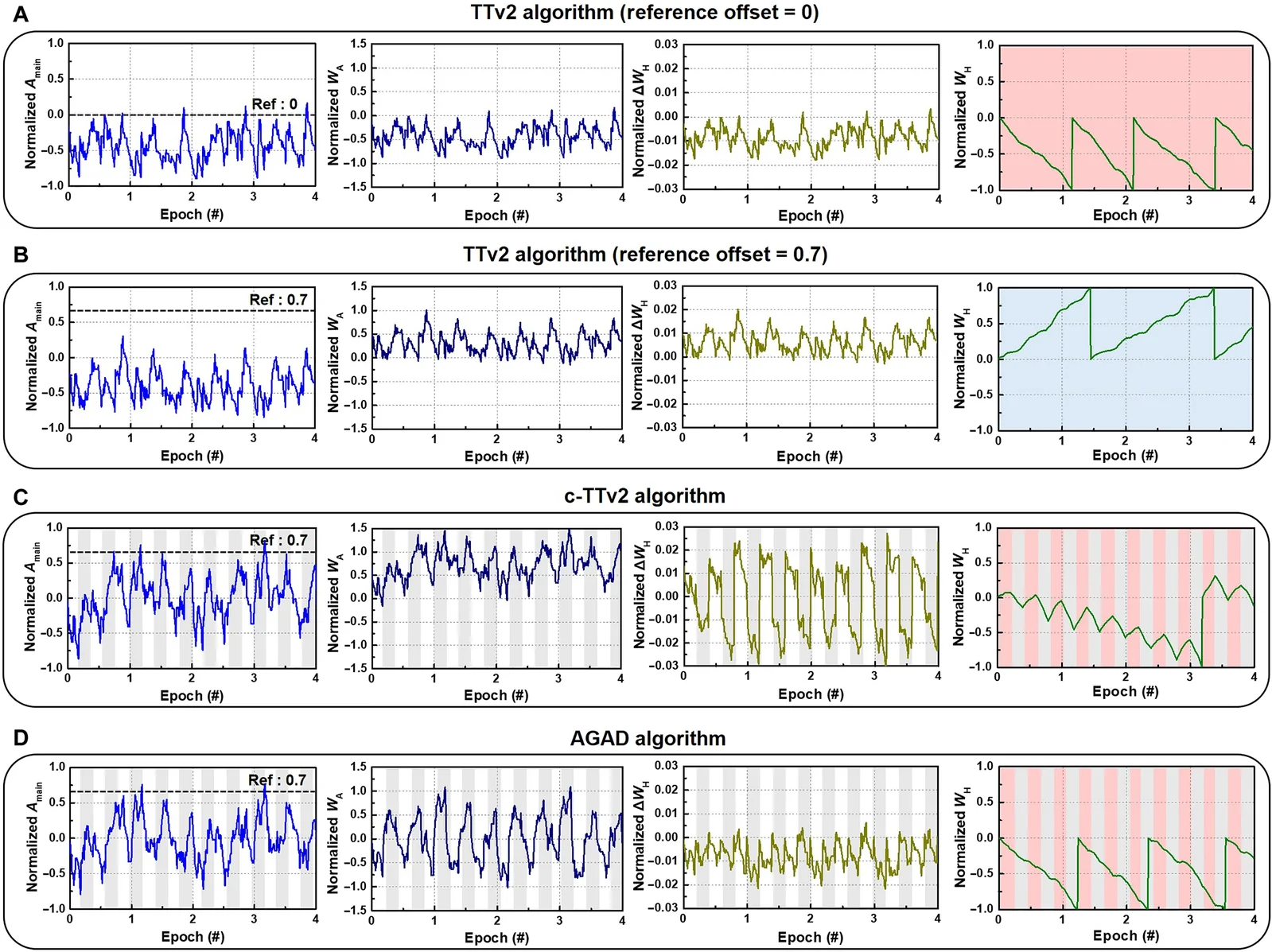
Comparative analysis through simulation of chopper technique and dynamic digital reference on weight updates in auxiliary and hidden arrays. Traces of the analog gradient *A*_main_, the *A* matrix weight *W*_A_, hidden weight change Δ*W*_H_, and hidden weight *W*_H_ under the following conditions: (**A**) TTv2 algorithm with the SP accurately defined at the weight-zero position. (${\overset{ˇ}{r}}_{\text{ij}}={\overset{ˇ}{a}}_{\text{ij}}^{*}$). (**B** to **D**) TTv2, c-TTv2, and AGAD algorithms with reference defined at a nonzero position. (${\overset{ˇ}{r}}_{\text{ij}}={\overset{ˇ}{a}}_{\text{ij}}^{*}-0.7$)

Figure 2 provides a comparative analysis of the chopper’s operation and its mechanism under different conditions: (Fig. 2B) no chopper (TTv2) with a correctly predefined reference (${\overset{ˇ}{r}}_{\text{ij}}={\overset{ˇ}{a}}_{\text{ij}}^{*}$), (Fig. 2C) no chopper (TTv2) and with a reference offset (${\overset{ˇ}{r}}_{\text{ij}}={\overset{ˇ}{a}}_{\text{ij}}^{*}-0.7$), (Fig. 2D) chopper (c-TTv2), and E. AGAD with a reference offset. Here, ${\overset{ˇ}{a}}_{\mathrm{ij}}^{*}$ denotes the SP, corresponding to the reference at the zero-value point of normalized weight without any offset. The dataset consists of 100 data points based on a simple scenario in which the activations are defined as x=−X, and the gradient inputs are given by d=αX+(1−α)Y, where X,Y∼N(0,1) are Gaussian random variables. In Fig. 2A, when the reference value of *A* matrix is set at the point where the weight is zero, the actual gradient *W*_A_ transferred to *H* matrix is updated in negative direction. The change among entering the hidden weight is also negative, resulting in a hidden weight *W*_H_ update change accumulation occurring at the negative threshold of −1. The example shown in Fig. 2B, under a reference setting where the weight is nonzero, the effective gradient value on the *H* matrix predominantly takes positive values, leading to weight updates in the unintended direction.

As depicted in Fig. 2C, we demonstrate through simulation that the problem can be resolved by incorporating the chopper technique into the gradient accumulation. The sign of the chopper flips based on the chopper period, which is the inverse of the chopper probability, and when the chopper’s sign is negative (i.e., within the gray region), the update of *A*_main_ matrix occurs in the opposite direction unlike Fig. 2B. Afterward, the reference is subtracted, and the original sign is recovered before the value is propagated to the *H* matrix, causing in the direction of the hidden weight change alternating between positive and negative values. Accordingly, even in the presence of a reference offset, the weight change in the negative direction becomes larger, inducing a downward accumulation in the H matrix, which corresponds to the correct direction of accumulation. Nonetheless, because of the presence of accumulation in the positive direction as well, the threshold is reached more slowly, introducing a drawback of slower learning. Figure 2D illustrates that, owing to the adjustable digital reference, the hidden weight change is predominantly negative, enabling rapid accumulation toward negative threshold even in the presence of an offset, as observed in Fig. 2B. It achieves the best performance among TT algorithms while significantly relaxing device requirements.

### Architecture and operation of 4T1R unit cell

**Fig. 3.**
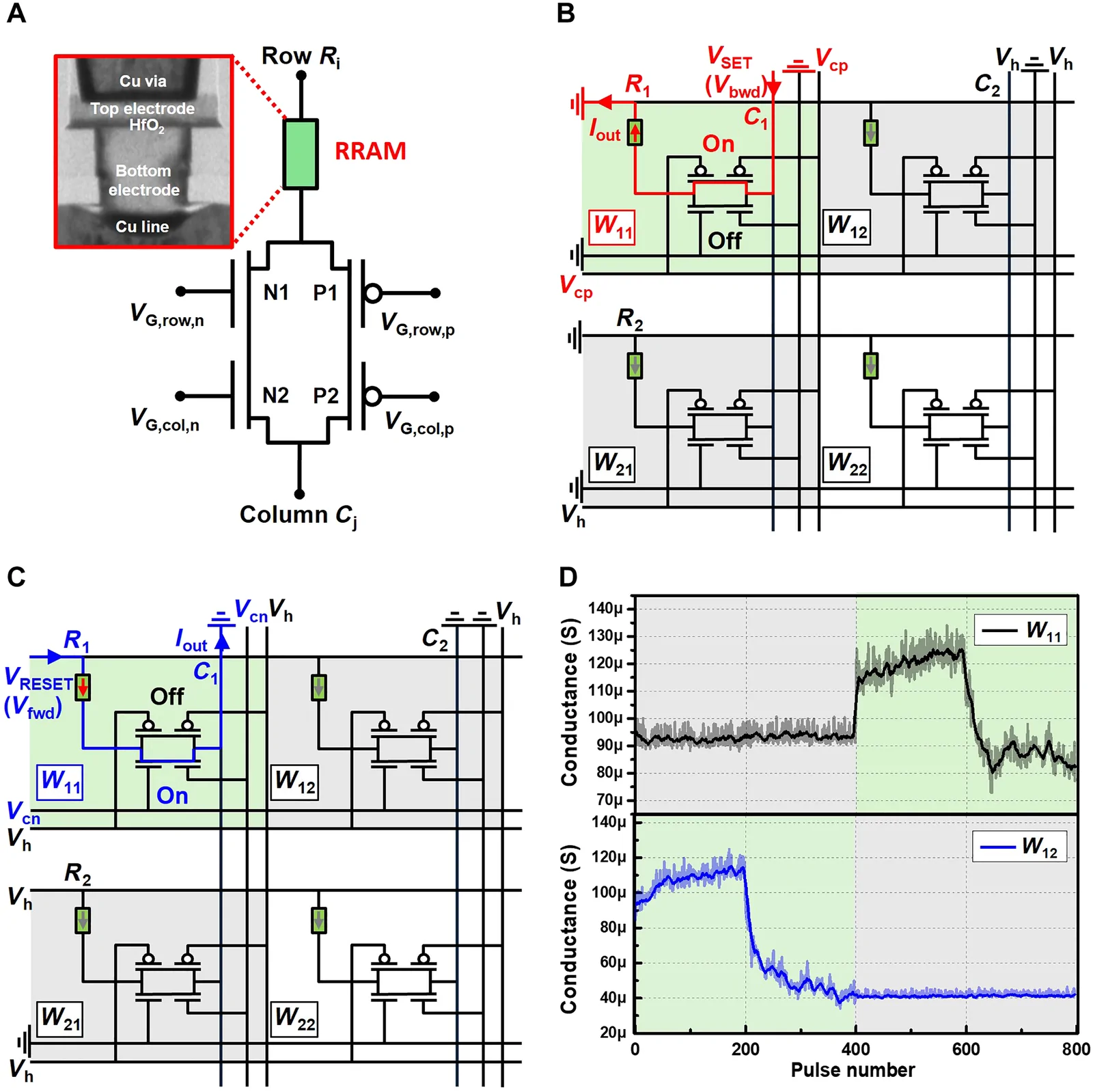
Operation principle of the unit cell with a 4T1R architecture and its array scalability. (**A**) Schematic of the 4T1R unit cell. Local selection scheme in a 2 × 2 crossbar array under (**B**) SET (negative programming) and backward read operations. (**C**) RESET (positive programming) and forward read operations. (**D**) Comparison of conductance switching between selected and half-selected cells.

Figure 3 describes the structure and functionality of the designed 4T1R unit cell. We design the 4T1R unit-cell integrates four transistors and an RRAM device, enabling adaptive adjustment of the compliance current (CC) based on the state of the RRAM. At the (*i*, *j*) position of the analog crossbar array, the 4T1R unit cell is configured as depicted in Fig. 3A: It comprises a single RRAM device located at the top and, at the bottom, two n-type field-effect transistors (NFETs), labeled N1 and N2, along with two p-channel field-effect transistors (PFETs), labeled P1 and P2. By applying voltage to the gate of each transistor in the unit cell, the CC of RRAM is precisely controlled during the forming, program, and read operation. Each row R_i_ or column in the crossbar array is connected to a current integrator, for measuring output, or to a logic input, for supplying voltage signals. As shown in the cross-sectional transmission electron microscope (TEM) image of Fig. 3A, the HfO_2_ based RRAM device is successfully integrated on a 130-nm complementary metal-oxide semiconductor (CMOS) technology node, which is compatible with back-end-of-line (BEOL) process.

The main operation of the 4T1R unit cell in 2x2 array can be distinguished by selectively turning both NFETs and PFETs on or off, as illustrated in Fig. 3 (B and C). In this architecture, PFETs are used during the SET (negative program) and backward read, while NFETs are activated for the RESET (positive program) and forward read. This design ensures directional control and enhances the accuracy of current readout. Furthermore, two identical transistors function as an AND gate, allowing current to flow only when both are turned on simultaneously. These operational characteristics highlight a key advantage of the proposed 4T1R structure over conventional 1T1R designs. While 1T1R cells suffer from limited selectivity and state-dependent read instability due to variations in $V_{\text{GS}}$, it ensures that the read current more accurately reflects the intrinsic device conductance, enabling more robust gating and precise control of current flow within the array. When the 4T1R unit cells are extended into a crossbar array, any specific cell can be locally selected and read by peripheral circuits. Each row and column lines are integrated with I/O interfaces that support analog, digital logic-in, and current-out for electrical interaction. In addition, selection drivers, NFET and PFET switches are deployed per row and column to control the on/off states at individual access points.

During the negative program, a program voltage (*V*_SET_) is applied to the target column line, and the corresponding row line is grounded to induce a negative bias across the RRAM. For backward read, a read voltage (*V*_bwd_) is applied to the selected column, causing the current *I*_out_ to flow in the row direction through the RRAM (Fig. 3B). Simultaneously, PFETs are turned on when the compliance voltage (*V*_cp_) is applied, and the NFETs are turned off by grounding gates. For the half-selected cells, either the row or the column line is not activated. One of the PFETs is turned off at high voltage (*V*_h_), and the cell is fully deactivated by the operation of the AND gate.

On the other hand, forming and positive program are conducted by supplying a voltage to the row and grounding the column. The forming process uses a higher forming voltage (*V*_form_) to trigger an initial thick conductive filament, and a program voltage (*V*_RESET_) is used to drive a positive bias across the RRAM. In the forward read, a read voltage (*V*_fwd_) is delivered to the row, the current flows in the column direction (Fig. 3C). To select desirable cell, NFETs are turned on when the compliance voltage (*V*_cn_) is applied, and the PFETs are turned off at *V*_h_. Likewise, in the half-selected condition as shown in Fig. 3B, either the row or the column line is deactivated, and one of the NFETs is grounded to turn the device off, completely isolating the cell.

Through the switching behavior of transistors, the 4T1R architecture enables accurate mode selection, ensuring functional versatility of the unit cell. Figure 3D shows this capability by comparing selected and half-selected cells in the array. In both cases, analog conductance modulation occurs solely within the selected cell, indicated by the green-shaded area. The half-selected cells, shown as gray-shaded areas, consistently exhibit no switching characteristics during peripheral circuitry activation. In addition, depending on the program direction, the selected cell exhibits clear potentiation and depression, revealing bidirectional synaptic behavior. Therefore, constructing the synaptic array using 4T1R unit cells mitigates the half-select problem, thereby minimizing write disturbances and preventing unwanted weight changes in adjacent cells during programming. The 4T1R unit cell design has significant potential for scaling into large synaptic arrays while minimizing undesired interference. The use of a 4T1R unit cell inherently incurs an area penalty compared to conventional 1T1R structures, as the unit cell footprint is primarily dominated by the access transistors rather than the resistive device itself, leading to an ∼4× increase in area. However, this overhead can be alleviated by leveraging advanced CMOS scaling with reduced forming voltage, which is compatible with 14-nm technology nodes, as demonstrated in 6T1R-based RRAM implementations (*42*). Furthermore, prior works on multitransistor memory cells have demonstrated scalability in advanced CMOS technologies and successful implementation of neural network tasks, suggesting that these architectures are not limited to a specific device type (*35*, *42*, *43*). Therefore, the proposed 4T1R structure can be extended to various memory technologies beyond RRAM.

### Electrical characterization of 4T1R unit cell and symmetry point analysis

**Fig. 4.**
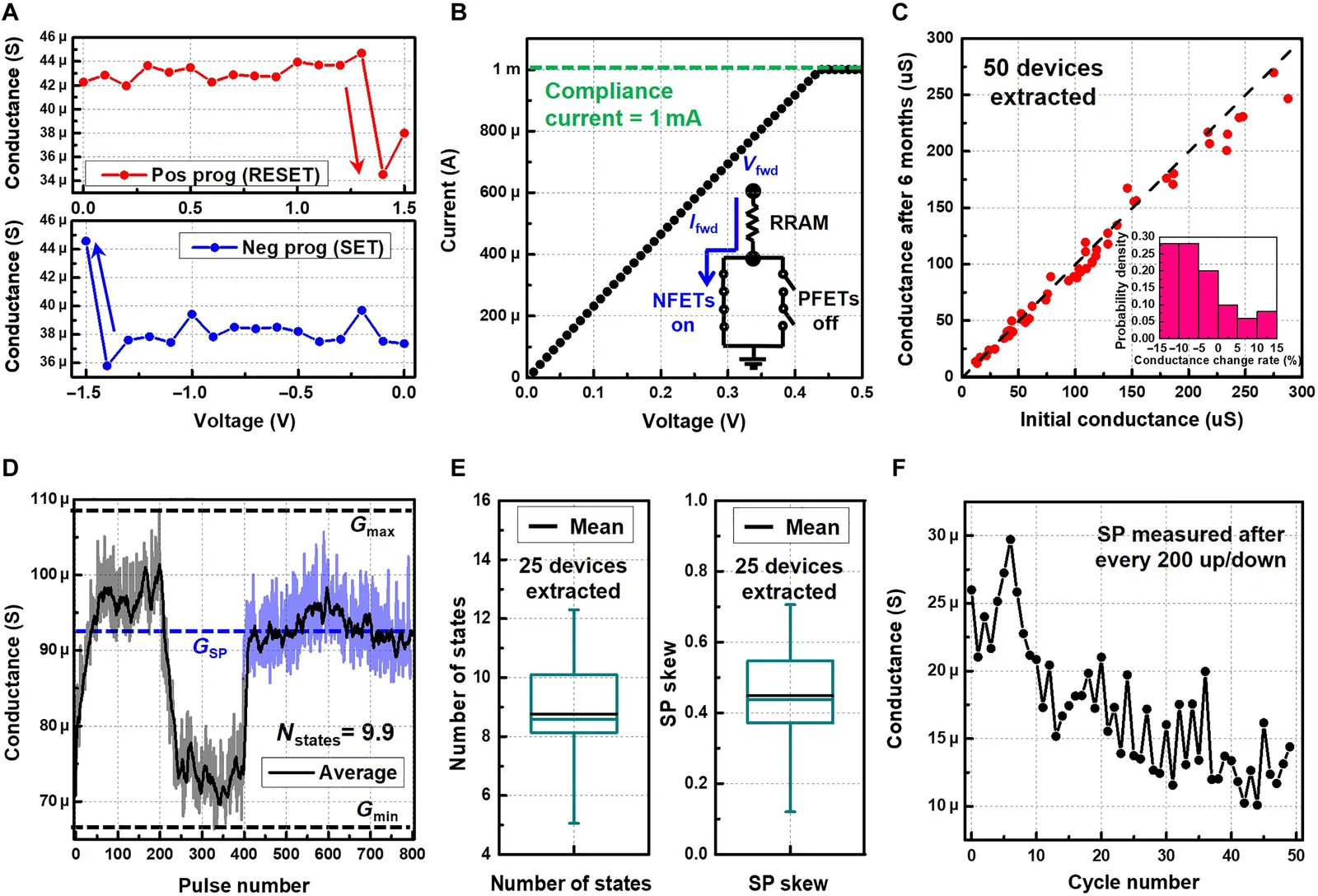
Electrical characterization of RRAM devices and SP. (**A**) Initial conditions of conductance programming during the RESET and SET operations. (**B**) Linear *I*-*V* characteristics in the LRS of RRAM device. (**C**) Six-month retention across 50 devices, plotted for cells showing less than 15% variation in conductance. (**D**) Representative potentiation/depression and SP characteristics of RRAM device. (**E**) Statistical analysis of the figure of merit obtained from 25 devices in the crossbar array. (**F**) Programmable reference variability with c-to-c shifts.

Figure 4 represents the electrical characteristics of the RRAM device unit, and extraction of its SP. To identify the precise threshold voltages that enable reliable control over the switching behavior of the device, Fig. 4A indicates the starting points of the conductance write process during the SET and RESET operations, which begin at −1.4 V for RESET and 1.5 V for SET, respectively. Figure 4B shows the current-voltage relation (*I*-*V*) characteristics of the RRAM device in the LRS after forming. The current increases linearly up to the CC of 1 mA, indicating that a sufficiently formed conductive filament enables ohmic conduction. Since conductance decay can compromise the retention of precise programmed values over extended training periods, it is essential to evaluate retention characteristics. Therefore, retention measurement conducted over a 6-month period, as shown in Fig. 4C, confirms the long-term reliability of RRAM based array, as validated across 50 devices. A dashed line connects the conductance values measured at the same point initially and after 6 months, most of the devices are distributed around the line. The inset graph shows the distribution of conductance change rate over a 6-month period. The highest portion of devices maintain their conductance, with only a minor decrease in conductance to below 15% after 6 months.

Figure 4D reveals the typical weight update characteristics of the RRAM device, exhibiting asymmetric conductance modulation. During the forming stage, a pulse with an amplitude of 3.5 V and a duration of 0.01 s is repeatedly applied five times. For the programming, 200 negative (−1.7 V) and positive (1.6 V) program pulses of width 320 ns are applied sequentially to the device. The device responds with bidirectional conductance modulation that follows the polarity of the potentiation, and depression pulses, which is sufficient for our demonstration. Moreover, the extracted SP serves to define the number of states (NOS; *N*_states_) and SP skew (SP_skew_) (*44*). NOS denotes the total number of distinguishable conductance levels within the switching window of the device. It is defined as follows

$$N_{\text{states}}=\frac{G_{\text{max}}-G_{\text{min}}}{\Delta \bar{G_{\text{SP}}}}$$ (2)

where the maximum and minimum conductance values obtained through 200 potentiation pulses and 200 depression pulses correspond to the maximum (*G*_max_) and minimum (*G*_min_) conductance of the device respectively, both of which are indicated by black dashed horizontal lines. $\Delta \bar{G_{\text{SP}}}$ is the average conductance change at the SP.

The SP skew indicates the relative location of SP between *G*_min_ and *G*_max_. It is defined as

$${\mathrm{SP}}_{\text{skew}}=\frac{G_{\text{max}}-\bar{G_{\text{SP}}}}{G_{\text{max}}-G_{\text{min}}}$$ (3)

where $\bar{G_{\text{SP}}}$ is the average conductance value at the SP.

Based on the above definitions, the NOS and SP skew value are statistically analyzed across 25 devices and visualized using box plot (Fig. 4E). The mean values are calculated as 8.8 for NOS and 0.45 for SP skew, which deviates from the optimal point of 50%, indicating that the skew is generally biased toward *G*_max_ in the crossbar array. Since the SP value is programmed as a reference, it is crucial that this point remains stable to ensure accurate weight readout. To further investigate the variability of the SP, the average value of the SP is repeatedly extracted after 200 potentiation and depression pulses. Figure 4F confirms that the SP position shifts with each cycle, implying that the reference point of weight cannot be fixed but subject to variation. Hence, it is essential to introduce a calibration method that continuously compensates for drifting reference. Figure S1 presents the configuration of the electrical testing setup used for characterizing the RRAM device, featuring the custom-designed RPU tester. This system can generate multiple parallel input patterns and measure output current through independent integrators. The setup also includes a semi-automatic probe station, a wafer, and a probe card.

### Operation mechanism of the AGAD algorithm on hardware

**Fig. 5.**
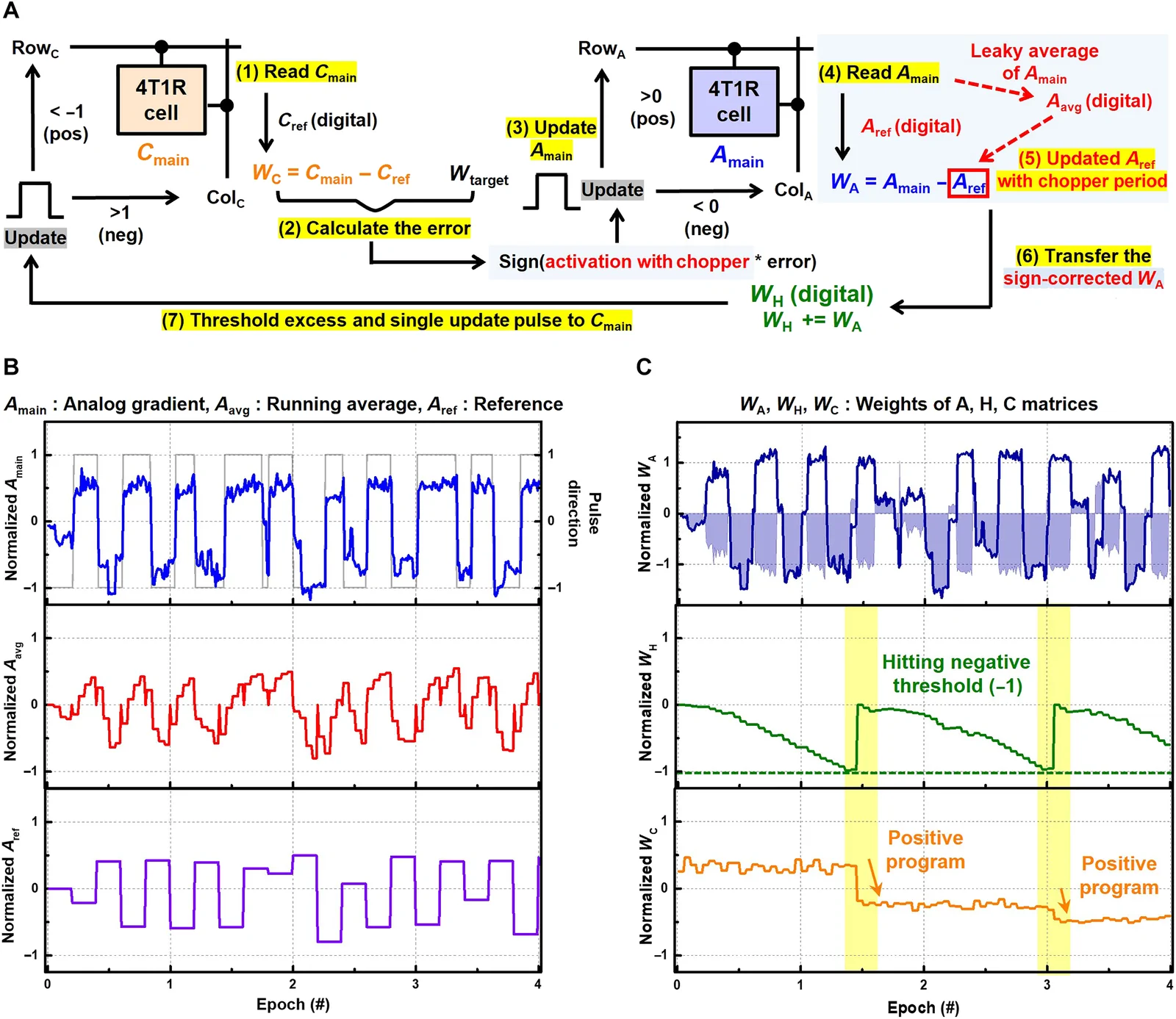
Hardware demonstration of the basic operational principle of the AGAD algorithm. (**A**) Diagram of the hardware configuration used for exploring the behavior of AGAD. (**B**) Traces of the analog gradient *A*_main_, the running average *A*_avg_, and the reference *A*_ref_ when negative direction weight update. (**C**) Traces of the A matrix weight *W_A_*, the hidden weight *W*_H_ and the *C* matrix weight *W*_C_. All traces correspond to weight updates in the negative direction.

Figure 5 highlights the basic working principle of the AGAD algorithm through experimental implementation using two-unit cells mapped to the analog gradient *A*_main_ and the analog weight *C*_main_ matrices, respectively. The input *x* is fixed at 1, and the constant error *d* is set to 0.25 to exemplify the most straightforward case for understanding the behavior. Figure 5A presents the hardware schematic designed for the demonstration of AGAD. The process begins by reading the *C*_main_ matrix to extract its weight, which is then used to calculate the error with respect to the target value. This calculated error serves as negative feedback guiding the subsequent update process. Critically, the sign of the outer product between the chopper-modulated activation, and the error determines the direction of the update: A positive sign results in an update pulse applied to the row line of *A*_main_ matrix, whereas a negative sign leads to an update pulse on the column. The sign change caused by chopper modulation at specific frequency drives gradient directions that switch between increasing and decreasing, as shown in Fig. 5B, where the update pulse direction, indicated by gray line, also changes accordingly. Simultaneously, a newly introduced digital running average *A*_avg_ matrix traces the *A*_main_ matrix based on a leaky integration, retrieving the value to be assigned to the reference *A*_ref_ matrix, and executing at a transfer period ns, as described by the following equation

$$A_{\text{avg}}=\left(1-\beta \right)A_{\text{avg}}+\beta A_{\text{main}}$$ (4)

where β is leaky-average time-scale, controlling how quickly the moving average *A*_avg_ adapts to new changes. A smaller β slows the tracking and produces a smoother average, while a larger β allows *A*_avg_ to quickly follow abrupt changes in *A*_main_. This moving average approach ensures reflecting the most recent state in the reference update process. Once updated, the value in *A*_avg_ matrix is reflected into the *A*_ref_ matrix in synchronization with the chopper probability, thereby continuously updating the reference with the recent value of *A*_main_. This new digital reference eliminates bias caused by reference mismatch, decoupling the gradient update behavior from offset and variation.

The actual trace held by *W*_A_ matrix is shown in Fig. 5C, and before being passed to the *H* matrix, the sign is recovered—as illustrated by the navy shaded area—so that most of the weight updates occur in the negative direction toward the correct directional threshold. When the value in the digital *H* matrix hits either the positive or negative threshold, an update pulse is delivered to the column or row line, respectively. In this case, since *W*_A_ value delivered to the *H* matrix is negative, the accumulated value moves toward the negative threshold by the transfer period. Whenever *W*_H_ reaches the negative threshold of −1, a negative program pulse is delivered to the *C*_main_ matrix, triggering in a new weight update in the negative direction accurately.

In the same manner, as shown in fig. S2, the capability of weight training in the positive direction is also experimentally confirmed. In this case, the sign of the error is inverted, demonstrating the feasibility of bidirectional weight updates using the AGAD on hardware. The A matrix operates in the opposite direction in fig. S2A, where the sign of *W*_A_ transferred to the H matrix is positive. Unlike in Fig. 5C, the positive threshold of +1 is hit, and the weight update proceeds in the positive direction. (fig. S2B)

### Array-level demonstration and cooptimization with AGAD algorithm

**Fig. 6.**
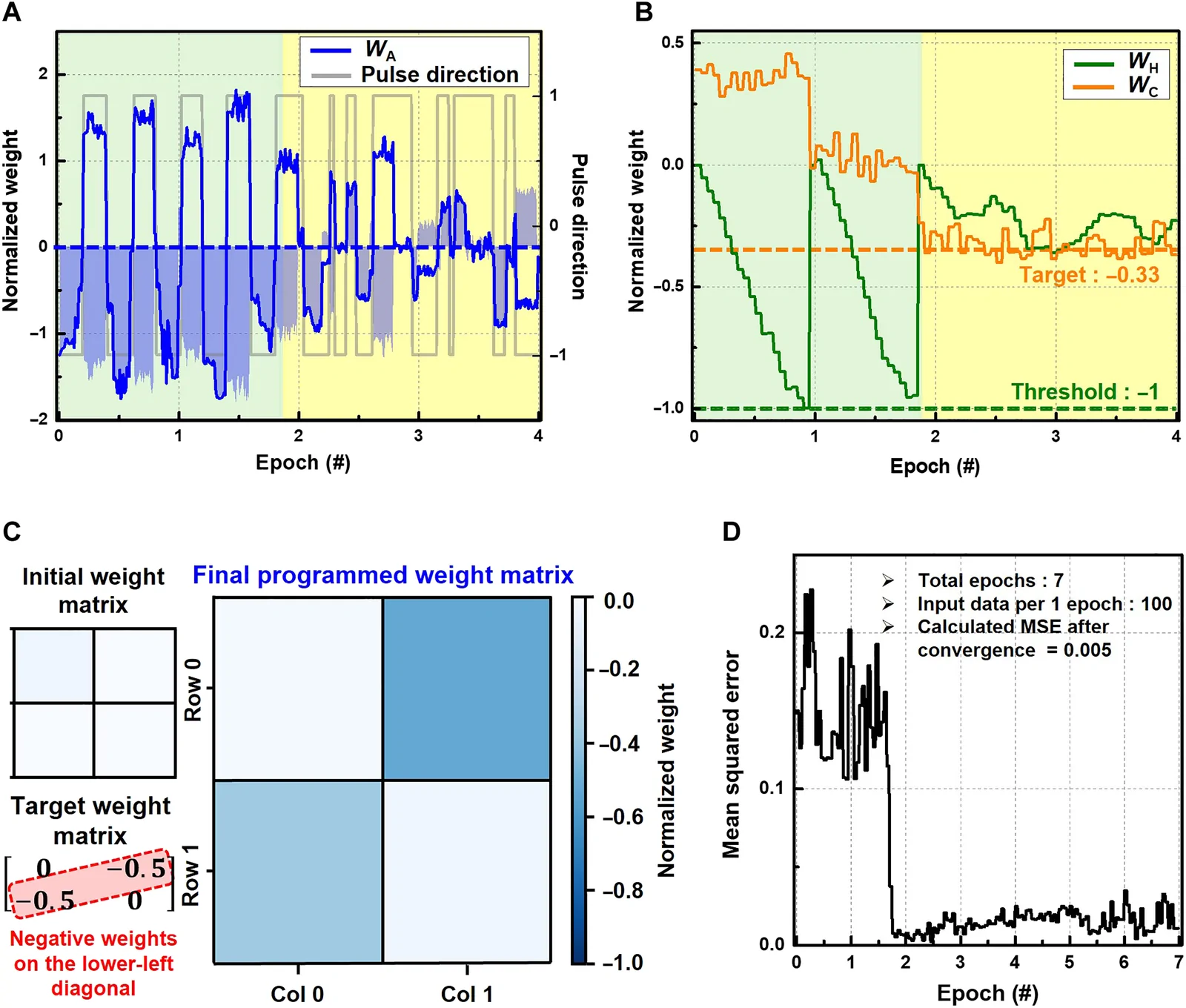
Experimental implementation of linear weight transfer training task using the AGAD algorithm. (**A**) Trace of *W*_A_. (**B**) Trace of *W*_C_. (**C**) Final programmed weight matrix of diagonal direction weight copying using two 2 × 2 arrays for *A* and *C* matrices. (**D**) Training accuracy based on the calculated MSE.

Figure 6 displays the experimental neural network training utilizing the AGAD on a RRAM array. The input dataset consists of 100 data points generated according to a simple linear relationship, where the target output y is computed as $y=w_{\text{target}}x+\sigma_{\text{noise}}$. The input *x* and target weight *w*_target_ are sampled from normal distributions with zero mean and variances of 1 and 0.3, respectively. For the dataset shown, the selected target weight is $w_{\text{target}}=-0.33$. Gaussian noise with a SD corresponding to 1% of the output range is added. In Fig. 6 (A and B), the traces of *W*_A_ and *W*_C_ during the weight transfer task are shown. Notably, the green regions that do not converge to the target weight indicate periods when the sign of the chopper, which directly maps to the pulse direction, changes periodically with every 20 updates. During this phase, the altering gradient is accumulated in the *A*_main_ matrix. When the effective gradient with sign recovery is transferred to the *H* matrix, it is delivered as a negative value, as visualized by the navy shaded area. Hence, the gradient accumulates in the *H* matrix in the negative direction, verifying stable convergence to the target weight even in RRAM array with small NOS values. Once the target weight is reached, the accumulated positive and negative gradients become statistically balanced regardless of the chopper probability. Thus, the directional update in the H matrix disappears, and no further weight update occurs.

Figure 6C further extends to a 2 × 2 array level integration, where both the *A* and *C* matrices are expanded to 2 × 2 arrays. In this configuration, the target is set to transfer a weight of −0.5 along the lower-left diagonal line only. This experimental result points up a robust implementation of the weight transfer task at the array level. It highlights that the device-algorithm cooptimization is accurately achieved without cross-talk between cells within the array, which is a critical requirement for scaling up to large-scale crossbar array, consistent with the observation in Fig. 3E. The corresponding training accuracy is quantitatively summarized in Fig. 6D. The mean squared error (MSE) between the transferred and target weights, serving as the training loss metric, is plotted. Low average MSE of 0.005 is achieved upon convergence to the target weight of −0.5, implying the high fidelity of the AGAD-based training even under realistic device conditions.

Figure S3 compares the hardware implementation performance between the c-TTv2 and AGAD. Figure S3A reveals that when the target weight is −0.5, the amount of weight update in the negative direction is greater in AGAD, proving its enhanced capability to drive convergence faster under the same conditions. Figure S3B further supports this, showing that the *H* matrix accumulates a larger amount of gradient in the negative direction, leading to faster convergence to the target weight. This is attributed to the use of dynamic reference accelerating the rate of gradient accumulation in AGAD, which allows fine-tuning of the update direction based on the moving average of the actual device response.

## DISCUSSION

We present the first hardware-level realization of AIMC training that uses gradient accumulation using the recently introduced chopper-based TT algorithm, AGAD. The hardware implementation leverages advantages of dynamic reference estimation strategy, which eliminates the need for preprogrammed reference arrays and differential readout circuits. This simplification substantially reduces calibration requirements, making the approach highly attractive for scalable crossbar array designs. On the input side, digital activations are converted into analog voltages or pulse trains via digital-to-analog converters (DACs) and applied through row and column drivers, while selective access to each 4T1R cell is controlled by switching transistors during computation and update phases. On the output side, column currents are accumulated based on Ohm’s and Kirchhoff’s laws, converted to voltage through integrators, and digitized by ADCs, which serve as the key interface between the analog and digital domains. Compared to conventional SGD-based architectures that rely on a single matrix for both weight storage and gradient computation, AGAD adopts a decoupled structure with two *A* and *C* matrices, enabling improved robustness against device nonidealities at the cost of additional control overhead. Notably, unlike prior TT variants that require an analog reference array for differential readout, AGAD uses a digital reference, allowing post-readout comparison in the digital domain.

In addition to circuit hardware efficiency, AGAD-based training can achieve the same time complexity, O(N), as TTv2 and c-TTv2, while preserving O (1) time complexity for forward and backward passes, along with O(N) overhead for dynamic reference estimation in *A*_avg_ matrix and computation in H matrix. The sign changes from chopper modulation contribute to negligible overhead and can be disregarded in time complexity estimation. Beyond that, AGAD demonstrates robustness to device asymmetry, as well as strong resilience to variations in reference offset and NOS (*39*). Thus, AGAD is more broadly applicable compared to SGD and prior TT-based approaches, supporting both highly asymmetric devices such as RRAM and more symmetric device technologies, including capacitive or ECRAM-based implementations. Furthermore, the reference offset in devices is not static but varies with time and programming cycles. AGAD operates reliably regardless of variations, offering versatility and suitability for feasible AIMC. As reported in previous studies, DNN training simulations under nonideal device conditions consistently show that AGAD achieves the lowest test error across various network architectures and variation scenarios (*39*, *42*). Consistent with this robustness, convergence behavior is further evaluated in this work. As shown in fig. S4, AGAD requires significantly fewer total transfer pulses to reach convergence compared to TTv2 and c-TTv2 under reference variability, as evaluated using a small Vision Transformer model (∼4.6-M parameters) trained on CIFAR-10 under both noiseless and noisy (σ = 0.25) reference conditions. This demonstrates that AGAD improves convergence efficiency by minimizing unnecessary updates while maintaining correct gradient directions.

On the other hand, certain limitations and trade-offs have been identified. The dynamic reference estimation accuracy is sensitive to performance of analog *A*_main_ matrix. Drift and high noise conditions may impair the stability of target weight convergence or slow training performance. Low endurance associated with frequent gradient accumulation can also accelerate device degradation due to repeated programming cycles. This degradation may reduce the precision of conductance updates, ultimately leading to cumulative gradient errors. While AGAD reduces analog hardware complexity relative to TT variants, it needs increased digital overhead for moving average computation. As digital resources grow in AIMC, low latency and high energy efficiency can be diminished by the increased digital overhead and data movement between analog and digital domains.

In terms of scalability, both analog and digital components scale with array size *N*. However, digital blocks further depend on bit precision *b*, resulting in an area scaling of *N*^2^ • *b*. This additional precision requirement increases memory footprint and associated digital logic overhead, including adders and accumulators for moving average updates; register arrays for storing the *H* matrix and reference values; and control logic for iterative update scheduling and data synchronization. For example, a representative 256 × 256 array with six-bit precision is considered. Under this configuration, the total number of array elements is *N*^2^ = 65,536, resulting in a digital scaling factor of *N*^2^·*b* = 65,536 × 6. The moving-average computation requires a corresponding number of accumulators and adders operating on *N*^2^ elements, The *H* matrix, moving-averaged values, and reference values must be stored in dedicated register arrays with six-bit precision, introducing an additional memory footprint that also scales with *N*^2^. In addition, control logic for iterative update scheduling and data synchronization must scale with the number of array elements, further increasing the complexity of the digital backend. As the array size increases, these factors collectively contribute to peripheral circuit complexity and routing overhead, particularly due to the dense interconnections required for bidirectional data transfer between analog array and digital computation units for realistic AGAD implementation. The need to individually access and control multiple transistors per 4T1R unit cell at the array-level introduces more sophisticated driving schemes and expanded control logic, increasing design complexity at the periphery. Moreover, the iterative digital reference updates introduce non-negligible update latency, which becomes more pronounced in large-scale arrays due to increased signal propagation delay and synchronization overhead.

From an energy consumption perspective, the total consumption can be divided into analog and digital components. While the analog core primarily accounts for VMM operations and peripheral circuitry, the digital core involves frequent memory access and iterative updates for dynamic reference estimation. As a result, the digital energy overhead becomes a dominant factor in AGAD due to accumulation and feedback operations. Nevertheless, AGAD still provides significant advantages over fully digital training approaches. It maintains substantially lower runtime owing to its linear computational complexity, achieving ∼50× lower latency compared to mixed precision, which suffers from quadratic scaling and results in significantly higher computational burden (*39*). Notably, this improvement is achieved without sacrificing accuracy, as AGAD attains a comparable error rate to the digital baseline, with only ∼2% test error (*39*).

In summary, this study highlights the first hardware implementation of AGAD on 130-nm CMOS technology–based RRAM array for analog neural network training. To understand the effect of chopper technique in detail, training simulations with chopper technique perfectly corrects weight update direction errors caused by inaccurately programmed reference values. A 4T1R unit-cell crossbar array is designed and fabricated for precise selection without half-select disturbances of adjacent cells, maintaining excellent 6-month retention characteristics. In addition, the moving-average–based digital reference in AGAD accelerates target weight convergence by accumulating larger gradient compared to c-TTv2. The superiority of AGAD is further evidenced by hardware demonstration of its operation mechanism and array-level neural network training, achieving a low MSE loss of 0.005 even under constrained device conditions. Our findings indicate that AGAD can significantly enhance training performance and applicability in future AIMC systems.

## MATERIALS AND METHODS

### Device fabrication

The RRAM stacks were integrated between two Cu BEOL levels of a 130 nm CMOS technology, as shown in Fig. 3A. The peripheral circuits of the RRAM array were fabricated between the front-end-of-line and the M5 Cu level. A SiN layer was then deposited, and vias were opened. Subsequently, a TiN film was deposited into the vias by chemical vapor deposition to form embedded bottom electrodes. After planarization by chemical mechanical polishing, a HfO_2_ layer was deposited using atomic layer deposition, followed by the deposition of the TiN top electrode via physical vapor deposition. The RRAM pillars were defined through reactive ion etching. Device integration was completed by forming contacts and depositing the final Cu level on top of the RRAM pillars.

### Simulation setup

During all simulations, a soft-bound synapse model was used to emulate the synaptic device behavior (*45*). Here, the average weight update change δ_w_ for a single update pulse when the weight is zero was set to 0.05, resulting in a NOS value of 40. The transfer period ns was set to 1, the learning rate of the *A* matrix λ_A_ was 1.0, and the learning rate for *H* matrix λ_H_ was 0.02. Both the positive and negative thresholds were uniformly set to 1 and −1, respectively. In the simulations for the c-TTv2 and AGAD, the chopper probability ρ was set to 0.05, the leaky average time-scale parameter β was set to 0.5, and the buffer scale parameter γ was set to 100.

### Measurement setup for electrical characterization

The electrical characterization of RRAM crossbar array was carried out at room temperature using RPU testing system, as shown in fig. S1. It features a high-speed switching network for rapid transition between bidirectional read and programming modes, which is essential for efficient characterization of memory devices. The RPU tester integrates several key functional blocks, including DACs, pattern generators, current measurement units, and analog-to-digital converters, allowing for various input signals and accurate readout of device responses. A personal computer is connected to the tester for system control, data acquisition, and real-time electrical characterization through the tester. The probe card makes direct contact with the wafer to apply input pulses generated by the pattern generator. The IV characteristics of the RRAM device in Fig. 4B were measured using the B1500A semiconductor parameter analyzer.

### Hardware demonstration setup of AGAD

The experimental demonstration of AGAD algorithm, shown in Figs. 5 and 6, was implemented using RRAM-based crossbar arrays to store the *A* and *C* matrices, while the *H* matrix was stored in digital memory. Weights were initialized using linear normalization based on the SP as a reference. The difference between *G*_max_ and *G*_min_ was used as the normalization factor to scale the conductance values into the range of [−1,1]. During all experiments, the chopper probability ρ and transfer period ns were set to 0.05 and 5, respectively. The leaky average time-scale parameter β was set to 0.5, and the buffer scale parameter γ was set to 50. For training, the learning rate for *A* matrix λ_A_ was set to 1.0, and the learning rate for *H* matrix λ_H_ was 0.05. A threshold of −1.0 was applied to the *H* matrix for noise tolerance. To perform the weight update potentiation and depression of the *A* and *C* matrices, write pulses with amplitudes of −1.7 V and 1.6 V, respectively, and a pulse width of 320 ns were used. For reading the *A* and *C* matrices, a read pulse of 0.2 V with a width of 2.5 μs was applied.
