# Supplementary material for: Implementation of offset corrected AGAD algorithm on 130-nm CMOS technology–based RRAM array for analog neural network training
Source: Sci Adv. 2026 Sep 25;12(39):eaee7134. doi: 10.1126/sciadv.aee7134 (PMC13614373; doi:10.1126/sciadv.aee7134)
Supplement: Supplementary file 1 [file sciadv.aee7134_sm.pdf]

Science Advances
AAAS

Supplementary Materials for

Implementation of offset corrected AGAD algorithm on 130-nm CMOS technology–based RRAM array for analog neural network training

Jimin Lee *et al.*

Corresponding author: Takashi Ando, andot@us.ibm.com; Seyoung Kim, kimseyoung@postech.ac.kr

Sci. Adv. **12**, eaee7134 (2026)
DOI: 10.1126/sciadv.aee7134

This PDF file includes:

Figs. S1 to S4

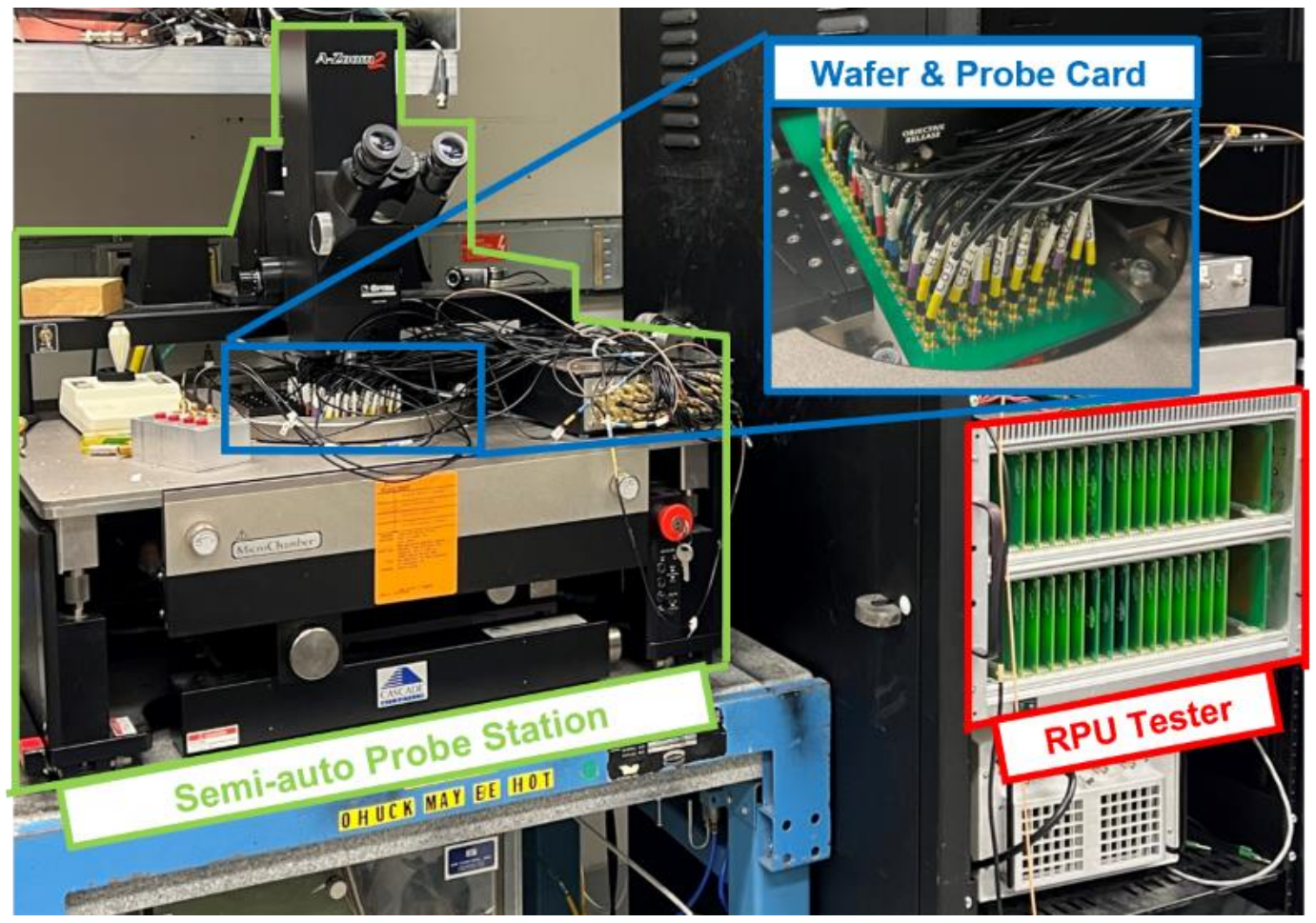

Fig. S1. Electrical testing setup incorporating RPU tester. RPU tester can generate multiple parallel input patterns for computation and measure output current through independent integrators. It also includes a semi-automatic probe station, a wafer, and a probe card.

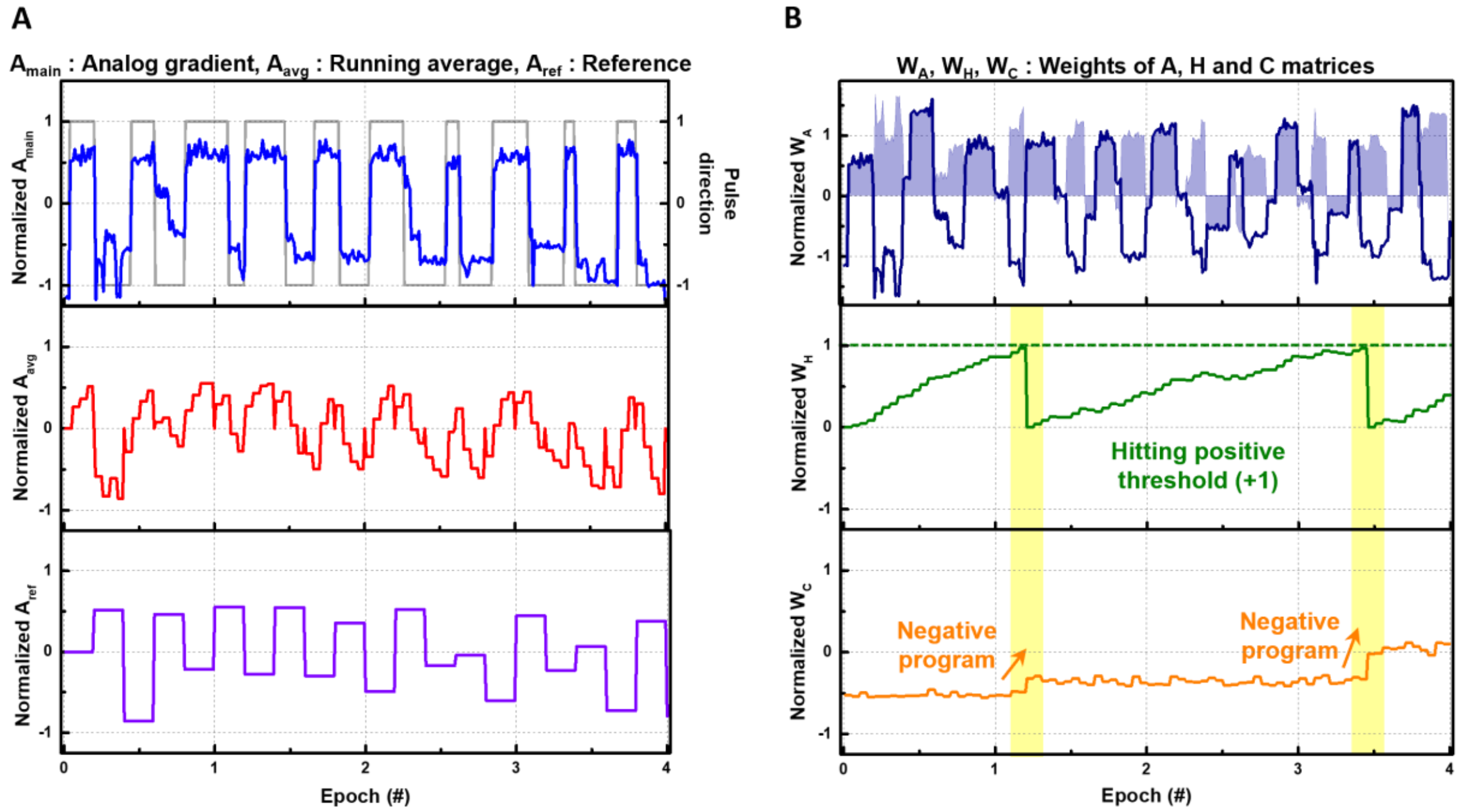

Fig. S2. Hardware demonstration of the basic operational principle of the AGAD algorithm. (**A**) Traces of the analog gradient A_{main}, the running average A_{avg}, and the reference A_{ref}. (**B**) Traces of the A matrix weight W_A, the hidden matrix weight W_H and the C matrix weight W_C. All traces correspond to weight updates in a positive direction.

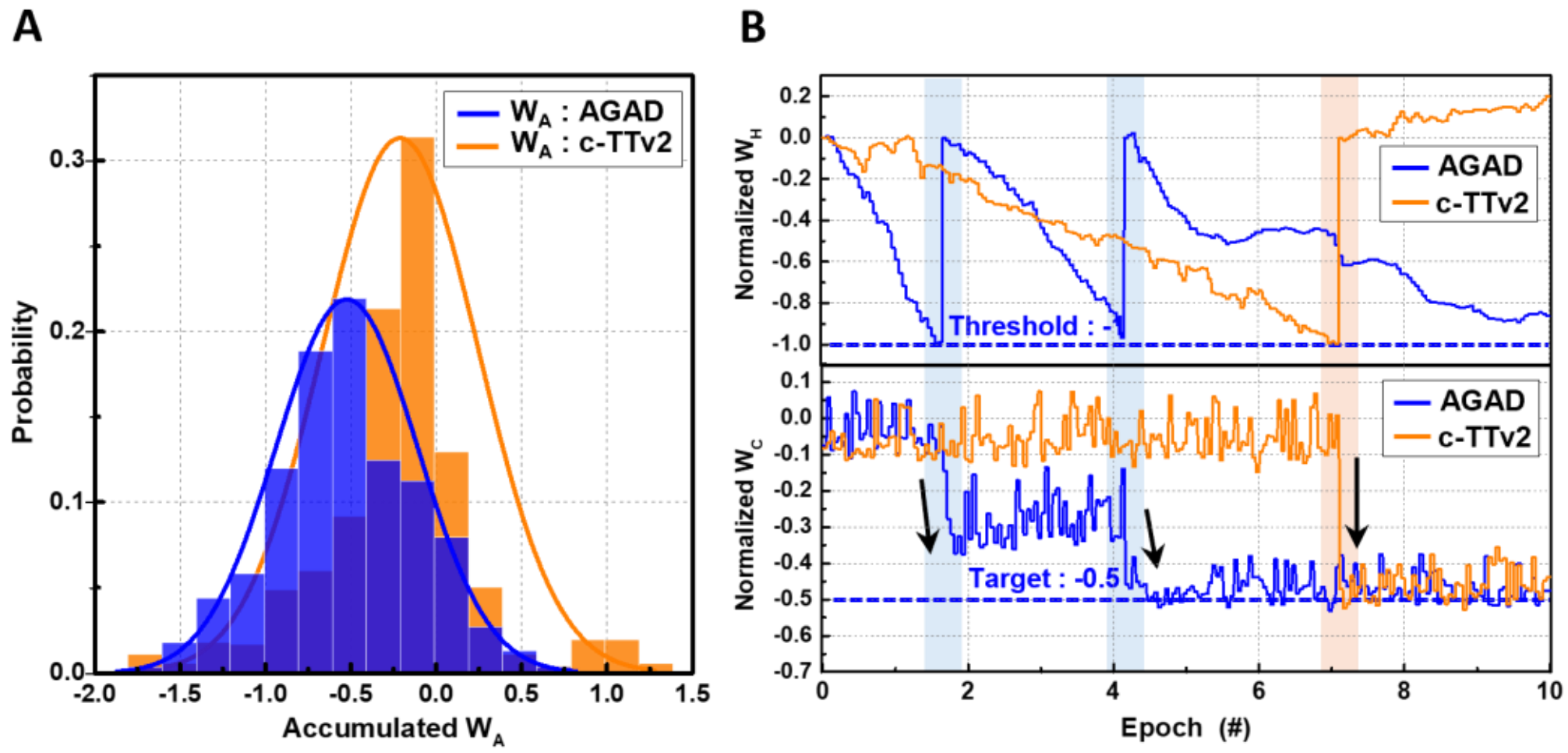

Fig. S3. Comparison of convergence between the c-TTv2 and AGAD algorithms during weight transfer training. (**A**) Distribution of W_A. (**B**) Traces of W_H and W_C. Target weight is -0.5 in both cases.

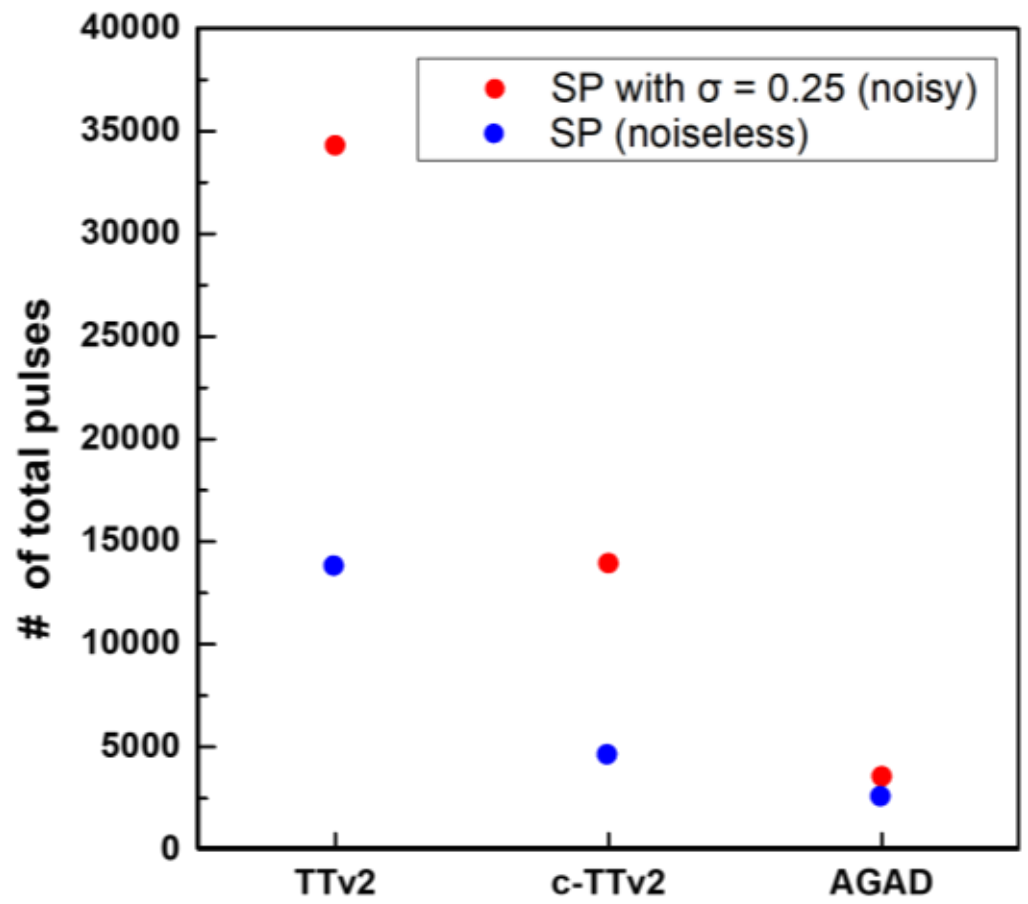

Fig. S4. Comparison of total transfer pulse counts for convergence across TTv2, c-TTv2, and AGAD. AGAD achieves convergence with significantly fewer transfer pulses than TTv2 and c-TTv2 in a small ViT model trained on CIFAR-10 under noiseless and noisy (σ = 0.25) reference conditions.
